# Circlize package in R and Analytic Hierarchy Process (AHP): Contribution values of ABCDE and *AGL6* genes in the context of floral organ development

**DOI:** 10.1371/journal.pone.0261232

**Published:** 2022-01-21

**Authors:** Gangxu Shen, Wei-Lung Wang

**Affiliations:** 1 School of Chinese Medicine for Post-Baccalaureate, I-Shou University, Kaohsiung, Taiwan; 2 Department of Biology, National Changhua University of Education, Changhua, Taiwan; Birla Institute of Technology and Science, INDIA

## Abstract

The morphological diversity of floral organs can largely be attributed to functional divergence in the MADS-box gene family. Nonetheless, research based on the ABCDE model has yet to conclusively determine whether the *AGAMOUS-LIKE 6* (*AGL6*) subgroup has a direct influence on floral organ development. In the current study, the ABCDE model was used to quantify the contributions of ABCDE and AGL6 genes in the emergence of floral organs. We determined that the flower formation contribution values of the ABCDE and *AGL6* genes were as follows: A gene, 0.192; B gene, 0.231; CD gene, 0.192; E gene, 0.385; and *AGL6*, 0.077. As *AGL6* does not directly influence floral structure formation, the contribution value of *AGL6* to flower formation was low. Furthermore, the gradient values of the floral organs were as follows: sepals, 0.572; petals, 1.606; stamens, 2.409; and carpels, 2.288. We also performed detailed analysis of the ABCDE and *AGL6* genes using the Circlize package in R. Our results suggest that these genes likely emerged in one of two orders: 1) B genes→CD genes→*AGL6*→E genes→A genes; or 2) B genes→CD genes→*AGL6*/E genes→A genes. We use the analytic hierarchy process (AHP) to prove the contribution values and gradient values of floral organs. This is the first study to understand the contribution values of ABCDE and *AGL6* genes using the AHP and the Circlize package in R.

## Introduction

The importance of MADS-box genes in the emergence of floral structures and subsequent morphogenesis makes them an ideal tool to examine the development of floral structures [1–3]. The nomenclature of this family is based on the members that were first identified: *MINICHROMOSOME MAINTENANCE 1* (*MCM1*) from *Saccharomyces cerevisiae*, *AGAMOUS* (*AG*) from *Arabidopsis thaliana*, *DEFICIENS* (*DEF*) from *Antirrhinum majus*, and *SERUM RESPONSE FACTOR* (*SRF*) from *Homo sapiens* [4,5]. It is generally presumed that an ancestor of the MADS-box gene developed prior to the evolution of eukaryotes, after which it evolved into two main clades, type I (*SRF-like*) and type II (*MEF2-like*) [6]. Among terrestrial plants, the structure of type II MADS-box transcription factors (TFs) comprise a MADS (M)-domain followed by an intervening (I), keratin-like (K), and C-terminal (C) domain (i.e., MIKC-type) [7,8]. MIKC-type TFs are further divided into MIKC*- and MIKC^C^ -type [9].

**Fig 1:**
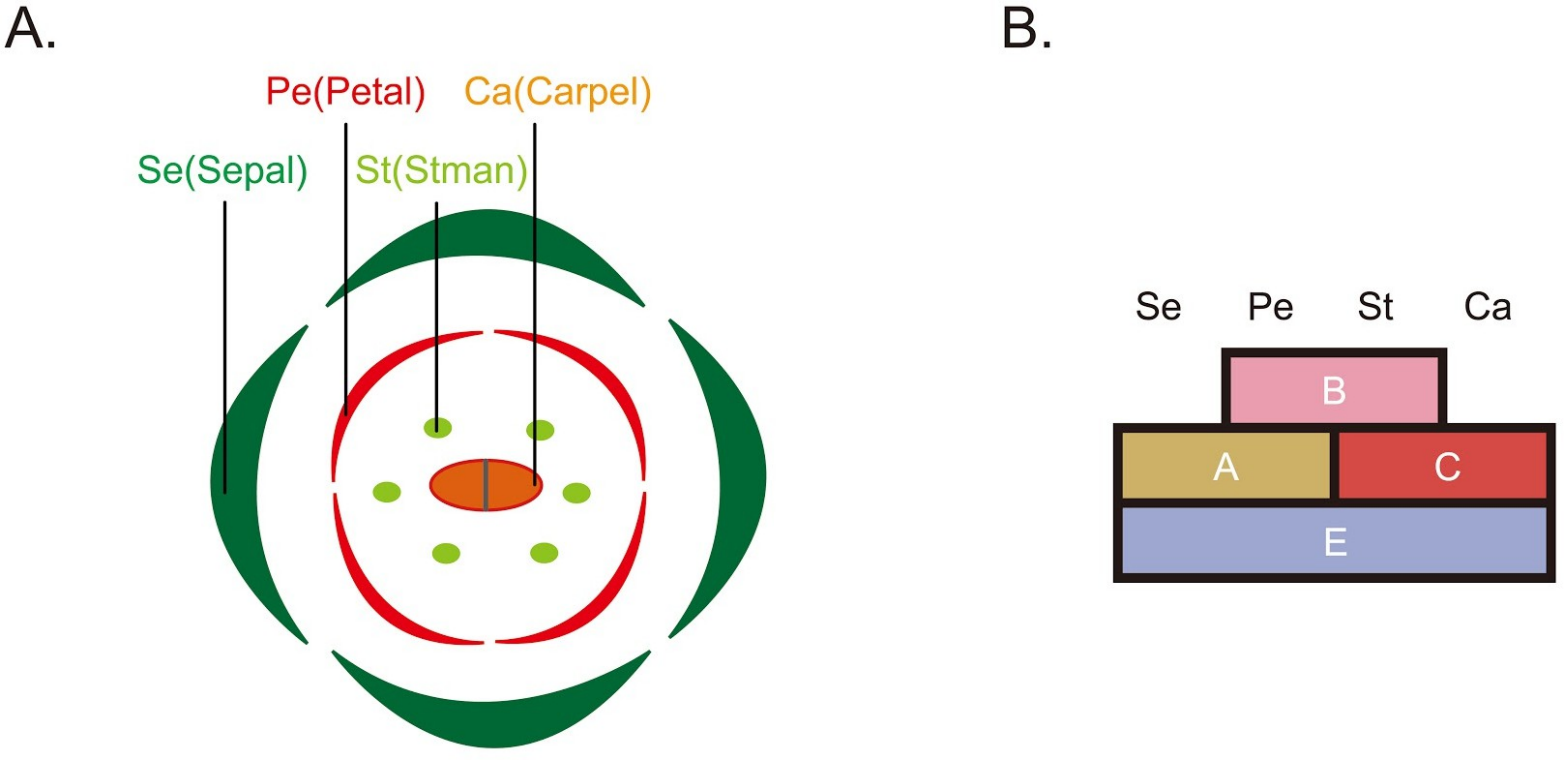
Top-down view of a flower and ABCDE model. Diagram A. top-down view of a flower. Se, sepal (green); pe, petal (red); st, stamen (green); ca, carpel (orange). Diagram B. A schematic of general flowers with four floral organs arranged in four floral whorls. The ABCDE model encompasses the original ABC model, which proposes that the A, B, and C genes function together to elaborate the different floral organs. Additionally, the ABCDE model proposes that E function is necessary to the formation of all organ types [11].

The ABCDE model posits that among MIKC^C^-type TFs, members of the ABCDE and *AGAMOUS-LIKE 6* (*AGL6*) subgroups play a key role in the development of floral organs (Fig 1) [3,5,10]. The ABCDE and *AGL6* genes form five subgroup clusters, namely *APETALA1* (*AP1* or A), *AP3/PISTILLATA* (*AP3/PI* or B), *AG/SHATTERPROOF/SEEDSTICK* (*AG/SHP/STK* or CD), *SEPALLATA* (*SEP* or E), and *AGL6/AGL13* (*AGL 6*). Essentially, A, B, and C proteins interact with E proteins in various combinations to form the various organ types [11]: sepals (A and E); carpels (CD and E); stamens (B, CD, and E); and petals (A, B, and E) [1,2,11–15]. Note that early researchers did not include E genes in sepals [16]. It is important to consider that E genes are involved in the formation of all floral organs [11], whereas *AGL6* genes are involved primarily in the formation of the flower and cone in seed plants [13,17]. *AGL6* and E genes present a high degree of sequence similarity and form sister clades in phylogenic trees [13]. It has been reported that *AGL6* genes in monocots and eudicots play an essential role in floral development [13,18]. The *AGL6*-like genes from grass form two paralogous clades: OsMADS17 and OsMADS6 [13,18]. The *Arabidopsis* genome contains two *AGL6* genes (*AGL6* and *AGL13*) [19], which suggests functional redundancy between the two genes.

**Fig 2:**
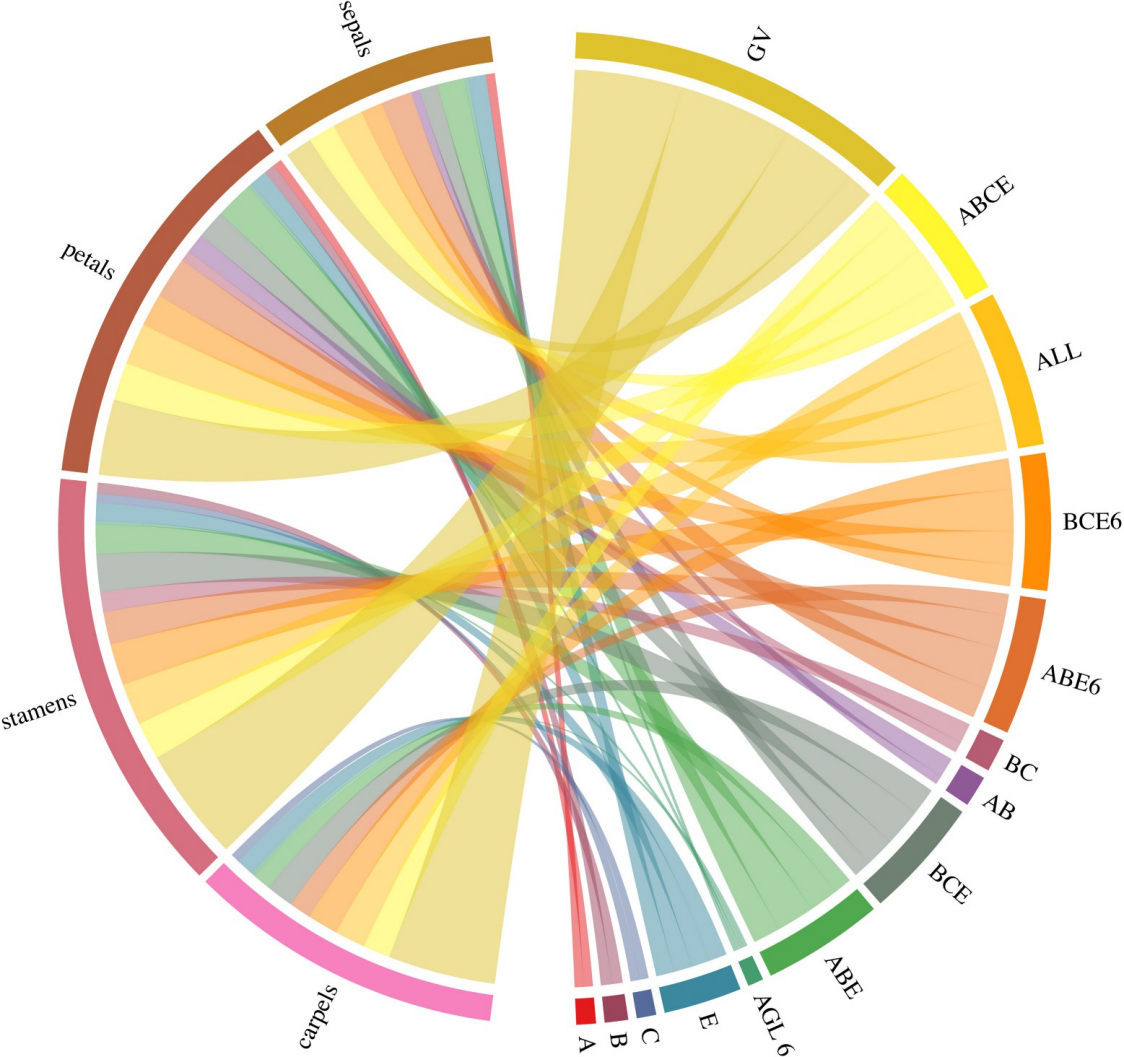
Example Circos plot created using the Circlize package in R, showing the contribution values of the ABCDE and *AGL6* genes to floral organ development, as derived using Bayesian tree and ABCDE models. GV: Gradient values (the gradient value of the floral organs refers to the product of the importance values of floral organs and the ABCDE flower formation contribution value); ABCE:A+B+C+E; ALL:A+B+C+E+*AGL6*; BCE6:B+C+E+*AGL6*; ABE6:A+B+E+*AGL6*; BC:B+C; AB:A+B; BCE:B+C+E; ABE:A+B+E.

Research into the origins of type II MADS-box genes has suggested that the B gene was the first to emerge [20–26]. The B/CD gene evolved relatively earlier than other flower identity genes [26]. However, related research has been unable to confirm the evolution order of *AGL6*/E/A gene. We used the ABCDE model (Fig 1) to estimate quantitatively the contribution of these genes to the development of floral organs. Furthermore, we performed detailed analysis of the ABCDE and *AGL6* genes using the Circlize package in R (Fig 2).

## Materials and methods

### Searching species databases

As shown in S1 Table, data pertaining to ABCDE and *AGL6* genes was obtained from the *Arabidopsis thaliana* Database (http://www.arabidopsis.org/) and *Oryza sativa* Database (http://rice.plantbiology.msu.edu/).

### Building alignment and phylogenetic trees

The amino acid sequences were aligned using the program MUltiple Sequence Comparison by Log-Expectation (MUSCLE) for treeconstruction using the program MEGA6. Initial trees were constructed using the BEAST2.2 to construct Bayesian phylogenies [28]. The BEAST analysis was performed using a JTT substitution model and Yule priors-model. The stationary distribution of the MCMC chains and the convergence of runs were monitored using Tracer (v. 1. 6) to determine the appropriate MCMC chain length such that the effective sample size of every parameter was larger than 200 as recommended. Tree pictures were generated using TreeAnnotator (v. 1. 8), with the first 1000 trees discarded as burn-in, and visualized using Figtree (v. 1. 4) [27].

### Circos plot

An initial plot was obtained using *circos*. *initialize or circos*.*initializeWithIdeogram* for the assignment of various categories of data to different sectors. We then used *circos*.*trackPlotRegion* to create regions for the plotting of new tracks and the inclusion of basic graphics. When drawing multiple tracks, this second step is repeated before using Circos to generate the image [28].

## Results and discussion

### Phylogenetic analysis of the ABCDE and *AGL6* genes

**Fig 3:**
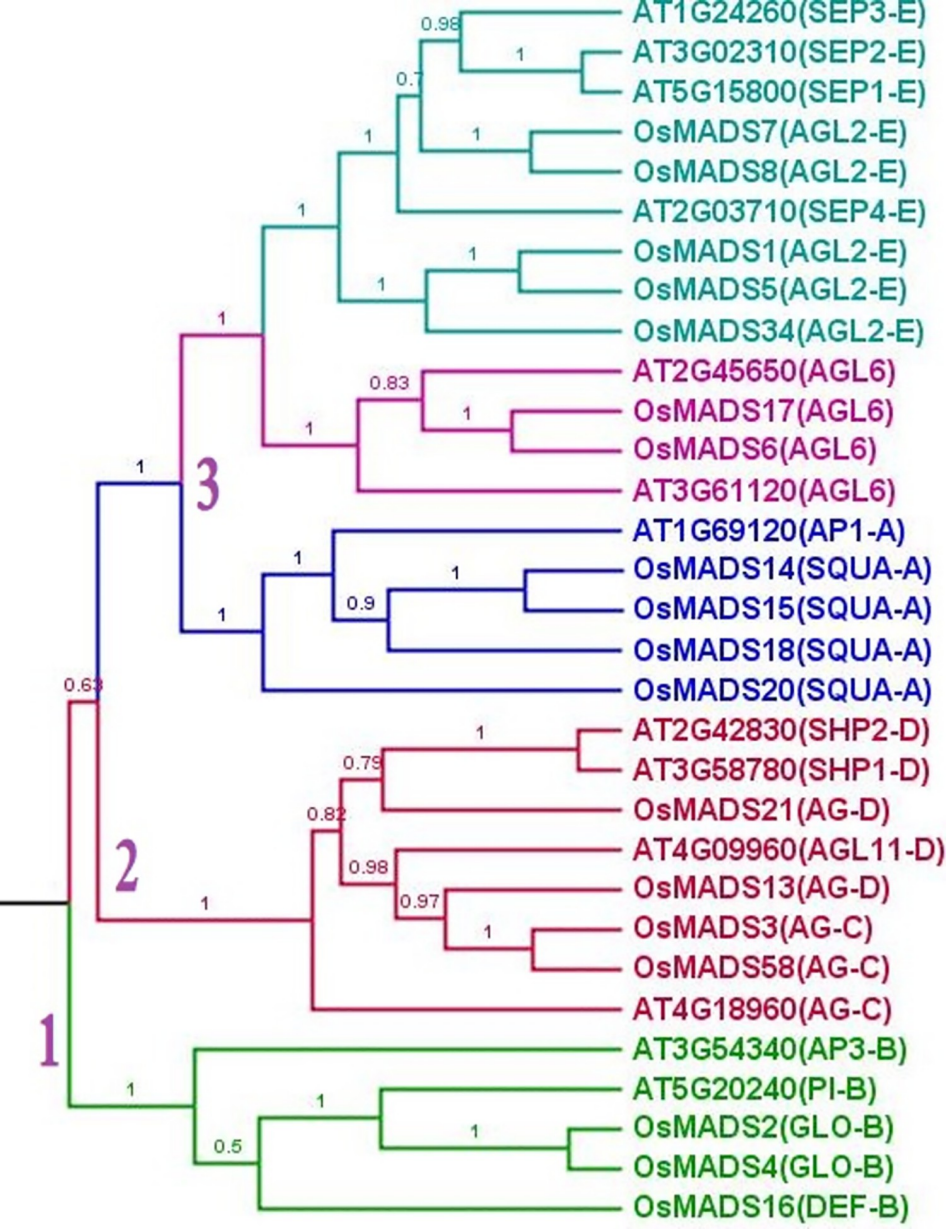
Phylogeny of the ABCDE and *AGL6* genes from *A*.*thaliana* and *O*. *sativa* and 31 classified protein sequences obtained using BEAST. The genes are indicated as follows: A gene, blue; B gene, green; CD gene, red; E gene, green; *AGL6*, pink. The Bayesian posterior probability values in tree. By using the BEAST tool, ABCDE and *AGL6* genes appeared in the following order: B gene→CD gene→*AGL6*/E/A gene. The numbers 1, 2, 3 represent the order of evolution of ABCDE and *AGL6* genes.

Bayesian methods were used to elucidate phylogenetic relationships (Fig 3) among the 31 sequences based on ABCDE and AGL6 protein sequences from *A*. *thaliana* and *O*. *sativa* (S1 Table). From Fig 3, the order of appearance was as follows: B gene → CD gene → *AGL6*/E/A gene. This result was followed to infer the contribution values of the ABCDE and *AGL6* genes and the gradient values of the floral organs.

We employed the Bayesian evolutionary analysis by sampling trees (BEAST) program to construct a phylogenetic tree (Fig 3) for use in illustrating the evolutionary relationship among all of the ABCDE and *AGL6* gene sequences. The Bayesian methods makes it possible to implement complex models of gene evolution [29].

### The contribution values of the ABCDE and *AGL6* genes

Assuming that the ABCDE genes contribute equally to flower formation, we assigned a flower formation contribution value of 1 unit for each of these genes. Thus, according to the ABCDE model (Fig 1), 2 units (A+E) contribute to sepal formation; 3 units (A+B+E) contribute to petal formation; 3 units (B+C+E) contribute to stamen formation; and 2 units (C+E) contribute to carpel formation. To maintain a maximum sum value of 1, the contribution values were adjusted as follows: sepals (A+E) = 0.2; petals (A+B+E) = 0.3; stamens (B+C+E) = 0.3; and carpels (C+E) = 0.2.

**Fig 4:**
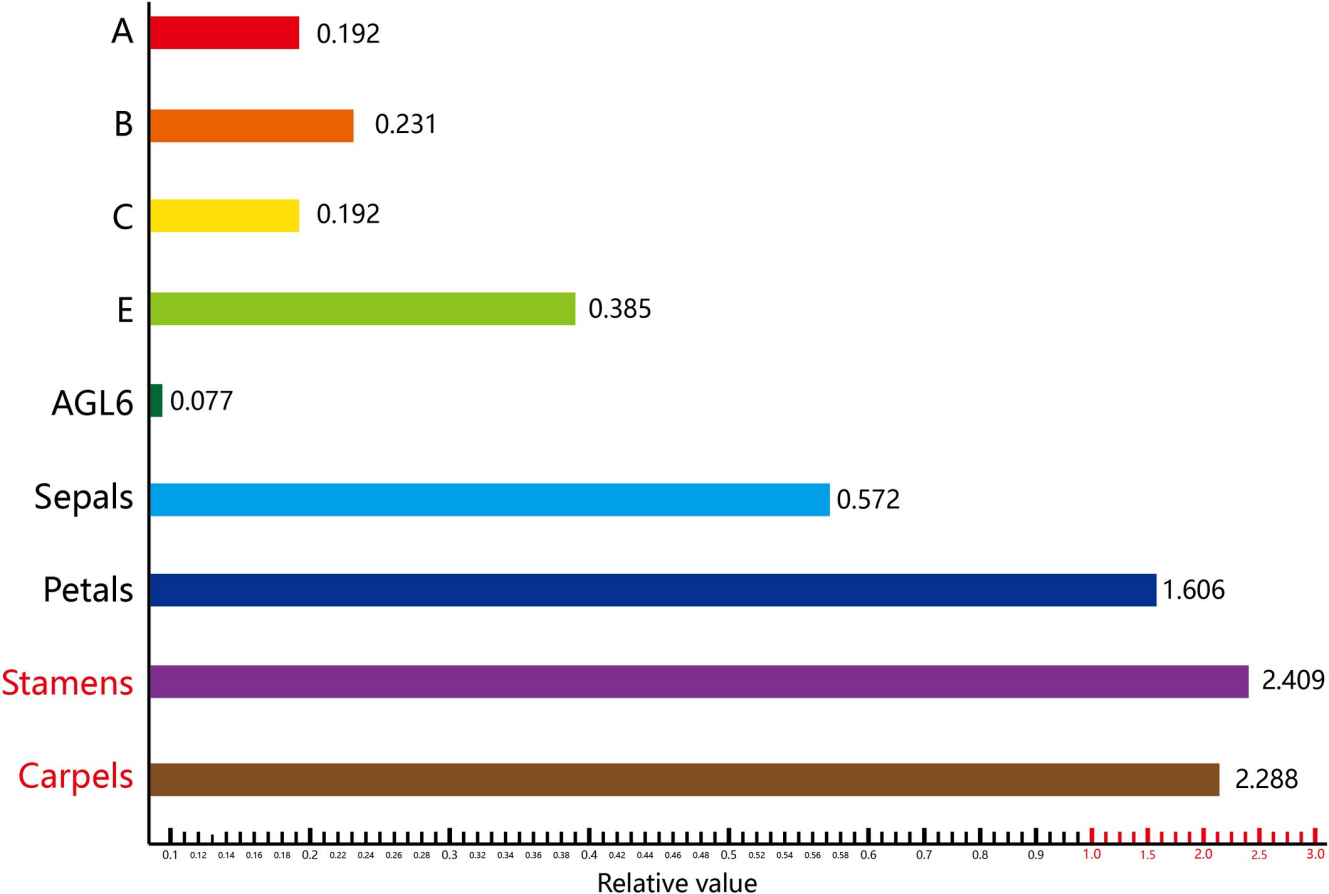
The contribution values of the ABCDE and *AGL6* genes and the gradient values of the floral organs. The flower formation contribution values of the ABCDE genes are as follows: A gene, 0.192; B gene, 0.231; CD gene, 0.192; and E gene, 0.385. The gradient value of the floral organs refers to the product of the importance values of floral organs and the ABCDE flower formation contribution value. The gradient values of the floral organs: 0.572; petals, 1.606; stamens, 2.409; and carpels, 2.288.

However, the contributions of the ABCDE genes should be adjusted based on the relative effects of their mutations on flower formation. As A gene mutants form only stamens and carpels [30], their flower formation contribution value is 0.5 (0.3+0.2), and their actual contribution value is 0.5 (1–0.5). As B gene mutants form only sepals and carpels [30], their flower formation contribution value is 0.4 (0.2+0.2), and their actual contribution value is 0.6 (1–0.4). As CD gene mutants form only sepals and petals [30], their flower formation contribution value is 0.5 (0.3+0.2), and their actual contribution value is 0.5 (1–0.5). Furthermore, as E gene mutants do not form flowers [30], therefore this gene has a flower formation contribution value of 1.0. According to the above assumptions, and given a maximum contribution sum value of 1, the flower formation contribution values of the ABCDE genes are as follows: A gene, 0.192; B gene, 0.231; CD gene, 0.192; and E gene, 0.385 (Table 1 and Fig 4).

**Table 1:** The contribution values of the ABCDE and *AGL6* genes.

	sepals	petals	stamens	carpels
A	0.192	0.192	0	0
B	0	0.231	0.231	0
C	0	0	0.192	0.192
E	0.385	0.385	0.385	0.385
*AGL6*	0.077	0.077	0.077	0.077
ABE	0.577	0.808	0.616	0.385
BCE	0.385	0.616	0.808	0.577
AB	0.192	0.421	0	0
BC	0	0.231	0.423	0
ABE6	0.654	0.885	0.693	0.462
BCE6	0.462	0.693	0.885	0.654
ALL	0.654	0.885	0.885	0.654
ABCE	0.577	0.808	0.808	0.577

ABE:A+B+E, BCE:B+C+E,AB:A+B,BC:B+C,ABE6: A+B+E+*AGL6*,BCE6: B+C+E+*AGL6*,ALL: A+B+C+E+*AGL6*,ABCE: A+B+C+E.

### The gradient values of the floral organs

Stamens produce pollen, which develops into male gametophytes [1]. Carpels develop in the center of the flower and produce ovules [1]. The presence of carpels unites angiosperms, making carpels the most important autapomorphy of the angiosperms [31]. The regenerative potential of stamens and carpels is only about half as high as that of petals [32]. Therefore, the regenerative ability of stamens and carpels is weaker, thereby increasing their importance. Sepals, which are sterile and green leaf-like organs, are the outermost organs of a flower (Fig 1), making them relatively unimportant.

Therefore, we were able to assume the relative importance of floral organs. From the innermost to outermost floral whorls, floral organs have relative importance values of 4 to 1. The assumption of this value is based on the reproductive importance of the flower and the regenerative ability of floral organs. Carpels are located in the innermost layer of the flower and are relatively more important than stamens. Therefore, considering the relative importance values, we set carpels to 4 and stamens to 3. The more important consideration is that the regenerative potential of carpels is half that of the petals [32], so petals were set to 2. Thus, the value of the floral organs is as follows: carpels (CD+E), (0.192+0.385)*4 = 2.288; stamens (B+CD+E), (0.231+0.192+0.385)*3 = 2.409; petals (A+B+E), (0.192+0.231+0.385)*2 = 1.606; and sepals (A+E), (0.192+0.385)*1 = 0.572 (Table 1 and Fig 4). Such a value is called a gradient value. The gradient value of the floral organs refers to the product of the importance values of floral organs and the ABCDE flower formation contribution value. The higher the gradient value of the organ, the earlier in evolutionary history it emerged. In summary, our analysis indicates that floral organs emerged in the following sequence: stamens, → carpels, → petals, and →sepals. The stamens and carpels are the reproductive structures of angiosperms, whereas petals and sepals are supporting structures that may attract pollinators but are not essential for reproduction.

B genes are associated with the formation of petals and stamens (in angiosperms) as well as male cones (in gymnosperms) [33,34]. Male cones contain microsporophylls in which male gametophytes (pollen) are produced. Female cones contain megasporophylls on the surface of which develop ovules [14]. The fact that sporophylls are the gymnosperm structures most closely related to carpels means that angiosperm flowers and gymnosperm cones could be regarded as homologous [14]. In angiosperms, *AGL6* genes are involved in flower development [13], whereas in gymnosperms, they are involved in cone formation [17]. Despite the fact that *AGL6* and ABCDE genes play equally important roles in the formation of these structures, it is important to consider the evolutionary order of the constituent genes. In Fig 3, The B/CD gene evolved relatively earlier than other flower identity genes [26]. The flower formation contribution value of the B/CD genes is 0.423 (0.231+0.192).

### Creating Circos plots of ABCDE and *AGL6* genes using the Circlize package in R

Circlize (version 4.0.2) was used to visualize within a circular layout the contribution values of ABCDE and *AGL6* genes in the context of floral organ development (Fig 2). Circos plots provide a basic template, which is easily modified with additional (higher-level) graphics focusing on specific details [28].

### Using the analytic hierarchy process (AHP) to prove the contribution values and gradient values of floral organs

The analytic hierarchy process (AHP) is a multiple criteria decision-making tool applicable to almost any situation that involves decision-making [35]. In accordance with the methods described in How to make a decision: The Analytic Hierarchy Process [Saaty, 36], we employed the following derivation (Tables 2–7):

**Table 2:** W1-W6 value.

	Sepals	Petals	Stamens	Carpels
Sepals	1	W1	W2	W3
Petals	$\frac{1}{W1}$	1	W4	W5
Stamens	$\frac{1}{W2}$	$\frac{1}{W4}$	1	W6
Carpels	$\frac{1}{W3}$	$\frac{1}{W5}$	$\frac{1}{W6}$	1

Wn = $\sqrt{W1\times W2}$.

W1 (Sepals-Petals) = $\sqrt{0.572\times 0.606}$ = 0.9584.

W2 (Sepals-Stamens) = $\sqrt{0.572\times 2.409}$ = 1.1738.

W3 (Sepals-Carpels) = $\sqrt{0.572\times 2.288}$ = 1.1440.

W4 (Petals-Stamens) = $\sqrt{1.606\times 2.409}$ = 1.9669.

W5 (Petals-Carpels) = $\sqrt{1.606\times 2.288}$ = 1.9169.

W6 (Stamens-Carpels_ = $\sqrt{2.409\times 2.228}$ = 2.3477.

**Table 3:** Sum of W1-W6 value.

	Sepals	Petals	Stamens	Carpels
Sepals	1	0.9584	1.1738	1.1440
Petals	1.0434	1	1.9669	1.9169
Stamens	0.8519	0.5084	1	2.3477
Carpels	0.8741	0.5216	0.4259	1
Sum	3.7694	2.9884	4.5666	6.4086

**Table 4:** A value divided by the sum.

	Sepals	Petals	Stamens	Carpels
Sepals	$\frac{1}{3.7694}$ = 0.2652	$\frac{0.9584}{2.9884}$ = 0.3207	$\frac{1.1738}{4.5666}$ = 0.2570	$\frac{1.1440}{6.4086}$ = 0.1785
Petals	$\frac{1.0434}{3.7694}$ = 0.2768	$\frac{1}{2.9884}$ = 0.3346	$\frac{1.9669}{4.5666}$ = 0.4307	$\frac{1.9169}{6.4086}$ = 0.2991
Stamens	$\frac{0.8519}{3.7694}$ = 0.2260	$\frac{0.5084}{2.9884}$ = 0.1701	$\frac{1}{4.5666}$ = 0.2190	$\frac{2.3477}{6.4086}$ = 0.3663
Carpels	$\frac{0.8741}{3.7694}$ = 0.2319	$\frac{0.5216}{2.9884}$ = 0.1745	$\frac{0.4259}{4.5666}$ = 0.0933	$\frac{1}{6.4086}$ = 0.1560

**Table 5:** V value.

	V value
Sepals	$\frac{0.2652+0.3207+0.2570+0.1785}{4}$ = 0.2554
Petals	$\frac{0.2768+0.3346+0.4307+0.2991}{4}$ = 0.3353
Stamens	$\frac{0.2660+0.1701+0.2190+0.3663}{4}$ = 0.2554
Carpels	$\frac{0.2319+0.1745+0.0933+0.1560}{4}$ = 0.1639

**Table 6:** A × V value.

	A × V Value							
Sepals	1	0.9584	1.1738	1.1440	×	0.2554	=	1.0641^a^
Petals	1.0434	1	1.9669	1.9169		0.3353		1.4183^b^
Stamens	0.8519	0.5084	1	2.3477		0.2554		1.0283^c^
Carpels	0.8741	0.5216	0.4259	1		0.1639		0.6708^d^

^a^ 1×0.2554+0.9584×0.3353+1.1738×0.2554+0.1440×0.1639.

^b^ 1.0434×0.2554+1×0.3353+1.9669×0.2554+1.9169×0.1639.

^c^ 0.8519×0.2554+0.5084×0.3353+1×0.2554+2.3477×0.1639.

^d^ 0.8741×0.2554+0.5216×0.3353+0.4259×0.2554+1×0.1639.

**Table 7:** [A] × [V]/[V].

	[A] × [V]/[V]
Sepals	$\frac{1.0641}{0.2554}$ = 4.1664
Petals	$\frac{1.4183}{0.3553}$ = 4.2299
Stamens	$\frac{1.0283}{0.2554}$ = 4.0262
Carpels	$\frac{0.6708}{0.1639}$ = 4.0927

* Average value (λ): $\frac{4.1664+4.2299+4.0262+4.0927}{4}$ = 4.1288.

CI (consistency index) = $\frac{\lambda -n}{n-1}=\frac{4.1288-4}{4-1}$ = 0.0429.

If the ratio of CI to that from random matrices is significantly small (specified at 10% or less), then we accept the estimate of w. Otherwise, we attempt to improve the consistency [35]. The CI in the current study was less than 0.1; therefore, we can infer that the contribution values of the floral organs are reasonable.

### A/E/*AGL6* genes arose through one of several possible evolutionary paths

#### *AGL6* evolved first among the *AGL6*/E/A genes

The flower formation contribution of the ABCDE genes to angiosperm stamens and carpels is 0.5 (0.2+0.3), and the angiosperm flowers and gymnosperm cones are homologous structures. The B/CD/*AGL6* genes and stamens and carpels evolved first; therefore, B+CD+*AGL6* = 0.5 and the contribution value of the *AGL6* gene is 0.077 (0.5–0.231–0.192). Although *AGL6* does not directly influence floral organ development, it is critical for the reproductive abilities of both gymnosperms and angiosperms [13,17]. Thus, it is not surprising that *AGL6* contributes less value to flower formation than do the ABCDE genes. The approach used here is one way to estimate the flower formation contribution value of *AGL6*; however, because *AGL6* does not directly influence floral structures, it is difficult to determine its flower formation contribution value.

a) The B/CD/*AGL6* genes evolved soon after the E genes.

As the B/CD/*AGL6*/E genes were available, flowers formed petals, stamens, and pistils, but not sepals.

b) The B/CD /*AGL6* genes evolved soon after the A genes.

As only the B/CD/*AGL6*/A genes were available, flowers could form due to the lack of the E gene. Therefore, a reasonable evolutionary order is B genes→CD genes→*AGL6*→E genes→A genes. *AGL6* and E genes have a high degree of sequence similarity and form sister clades in phylogenetic trees [32]. Another possible evolutionary order is: B genes→CD genes→*AGL6*/E genes→A genes.

### The E genes evolved first among the *AGL6*/E/A genes

a) The B/CD/E genes evolved soon after the *AGL6* gene.

As B/CD/E/*AGL6* genes were available, flowers formed stamens and pistils, but still lacked the A gene needed for sepals and petals.

b) The B/CD/E genes evolved soon after the A genes.

The presence of B/CD/E/A genes, but the absence of *AGL6*, may have resulted in defective flower development. Therefore, it is unlikely that the E genes evolved first among the *AGL6*/E/A genes.

#### The A gene evolved first among the *AGL6*/E/A genes

In the absence of E and *AGL6* genes, flower formation would not have been possible. Therefore, it is unlikely that the A gene evolved first among the *AGL6*/E/A genes.

## Conclusions

We use the analytic hierarchy process (AHP) to prove the contribution values and gradient values of floral organs. This is the first paper to understand contribution values of ABCDE and *AGL6* genes using the AHP. According to the proposed ABCDE model (Fig 1), the flower formation contribution values of the ABCDE and *AGL6* genes are as follows: A gene, 0.192; B gene, 0.231; CD gene, 0.192; E gene, 0.385; and *AGL6*, 0.077 (Fig 4). Furthermore, the following gradient values of the floral organs were calculated: sepals, 0.572; petals, 1.606; stamens, 2.409; and carpels, 2.288(Fig 4). The gradient value of the floral organs refers to the product of the importance values of floral organs and the ABCDE flower formation contribution value. Floral organs with a higher gradient value emerged earlier in evolutionary history. Hence, our analysis suggests that the order in which the floral organs evolved was stamens, carpels, petals, and then sepals. Additionally, the ABCDE and *AGL6* genes may have emerged in the following order: B genes→CD genes→*AGL6*→E genes→A genes. Another possible evolutionary order is B genes→CD genes→*AGL6*/E genes→A genes. We also performed detailed analysis of the ABCDE and *AGL6* genes using the Circlize package in R (Fig 2). This is the first study to use Circos plots of ABCDE and *AGL6* genes using the Circlize package in R. This research provides a refined model for floral organ evolution that can be used to explore the emergence of floral organs and the origin of the MADS-box genes.

## Supporting information

S1 Table The homeotic gene classification of *Arabidopsis thaliana* and *Oryza sativa*. (DOCX)
