# Supplementary material for: Circlize package in R and Analytic Hierarchy Process (AHP): Contribution values of ABCDE and AGL6 genes in the context of floral organ development
Source: PLoS One. 2022 Jan 21;17(1):e0261232. doi: 10.1371/journal.pone.0261232 (PMC8782415; doi:10.1371/journal.pone.0261232)
Supplement: S1 Table — (DOCX) [file pone.0261232.s001.docx]

**Table S1.**The homeotic gene classification of *Arabidopsis thaliana* and *Oryza sativa.*

| Class | *A. thaliana* | *O. sativa* |
| --- | --- | --- |
| A gene | *AT1G69120* | *OsMADS14*  *OsMADS15*  *OsMADS18*  *OsMADS20* |
| B gene | *AT3G54340*  *AT5G20240* | *OsMADS2*  *OsMADS4*  *OsMADS16* |
| CD gene | *AT2G42830*  *AT3G58780*  *AT4G09960*  *AT4G18960* | *OsMADS3*  *OsMADS13*  *OsMADS21*  *OsMADS58* |
| E gene | *AT1G24260*  *AT2G03710*  *AT3G02310*  *AT5G15800* | *OsMADS1*  *OsMADS5*  *OsMADS7*  *OsMADS8*  *OsMADS34* |
| AGL6 gene | *AT2G45650*  *AT3G61120* | *OsMADS6*  *OsMADS17* |
